# Quantum Trajectories: Real or Surreal?

**DOI:** 10.3390/e20050353

**Published:** 2018-05-08

**Authors:** Basil J. Hiley, Peter Van Reeth

**Affiliations:** Department of Physics and Astronomy, University College London, Gower Street, London WC1E 6BT, UK

**Keywords:** Stern-Gerlach, trajectories, spin

## Abstract

The claim of Kocsis et al. to have experimentally determined “photon trajectories” calls for a re-examination of the meaning of “quantum trajectories”. We will review the arguments that have been assumed to have established that a trajectory has no meaning in the context of quantum mechanics. We show that the conclusion that the Bohm trajectories should be called “surreal” because they are at “variance with the actual observed track” of a particle is wrong as it is based on a false argument. We also present the results of a numerical investigation of a double Stern-Gerlach experiment which shows clearly the role of the spin within the Bohm formalism and discuss situations where the appearance of the quantum potential is open to direct experimental exploration.

## 1. Introduction

The recent claims to have observed “photon trajectories” [1,2,3] calls for a re-examination of what we precisely mean by a “particle trajectory” in the quantum domain. Mahler et al. [2] applied the Bohm approach [4] based on the non-relativistic Schrödinger equation to interpret their results, claiming their empirical evidence supported this approach producing “trajectories” remarkably similar to those presented in Philippidis, Dewdney and Hiley [5]. However, the Schrödinger equation cannot be applied to photons because photons have zero rest mass and are relativistic “particles” which must be treated differently. In fact details of how to treat photons and the electromagnetic field in the same spirit as the non-relativistic theory have already been given in Bohm [6], Bohm, Hiley and Kaloyerou [7], Holland [8] and Kaloyrou [9], but this work seems to have been ignored. Flack and Hiley [10] have re-examined the results of the experiment of Kocsis et al. [1] in the light of this electromagnetic field approach and have reached the conclusion that these experimentally constructed flow lines can be explained in terms of the momentum components of the energy-momentum tensor of the electromagnetic field. What is being measured is the *weak value* of the Poynting vector and not the classical Poynting vector suggested in Bliokh et al. [11].

This leaves open the question of the status of the Bohm trajectories calculated from the non-relativistic Schrödinger equation [4,5] for particles with finite rest mass. The validity of the notion of a quantum particle trajectory is certainly controversial. The established view has been unambiguously defined by Landau and Lifshitz [12]:—“In quantum mechanics there is no such concept as the path of a particle”. This position was not arrived at without an extensive discussion going back to the early debates of Bohr and Einstein [13], the pioneering work of Heisenberg [14] and many others [15]. We will not repeat these arguments here.

In contrast to the accepted position, Bohm showed how it was possible to define *mathematically* the notion of a local momentum, p(r,t)=∇S(r,t), where S(r,t) is the phase of the wavefunction. From this definition it is possible to calculate flow-lines which have been interpreted as ‘particle trajectories’ [5]. To support this theory, Bohm [4] showed that under polar decomposition of the wave function, the real part of the Schrödinger equation appears as a deformed Hamilton-Jacobi equation, an equation that had originally been exploited by Madelung [16] and by de Broglie [17].

Initially this simplistic approach was strongly rejected as it seemed in direct contradiction to the arguments that had established the standard interpretation, even though the approach was based on the Schrödinger equation itself with no added new mathematical structures. However, recently this approach has received considerable mathematical support from the extensive work that has been ongoing in the literature exploring the deep relation between classical mechanics and the quantum formalism which has evolved from a field called “pseudo-differential calculus”. Specific relevance of this work to physics can be found in de Gosson [18] and the references found therein.

In this paper we want to examine one specific criticism that has been made against the notion of a “quantum trajectory”, namely the one emanating from the work of Englert et al. [19] (ESSW). They conclude, “the Bohm trajectory is here macroscopically at variance with the actual, that is: observed track. Tersely: Bohm trajectories are not realistic, they are surreal”. A similar strong criticism was voiced in Scully [20] who added that these trajectories were “at variance with common sense”. However the claim of an “observed track" in the above quotation should arouse suspicion coming from authors who claim to defend the standard interpretation as outlined in Landau and Lifshitz [12] .

The first part of the ESSW argument involved what they called the ‘standard analysis’ of a gedanken experiment consisting of several Stern-Gerlach magnets, an experiment that is discussed in Feynman [21]. It is this part of the argument that we examine in this paper. We show that they arrive at the wrong conclusion because they have not carried through the analysis correctly. Although Hiley [22] and Hiley and Callaghan [23] have presented a detailed criticism of this topic before in a different context, the point that we make in this paper is new. The standard use of quantum mechanics itself shows that what ESSW call the “macroscopically observed track” is identical to what has been called the “Bohm trajectory”. We support our arguments with detailed simulations of potential experiments that are being planned at present with our group at UCL.

## 2. Re-Examination of the Analysis of ESSW

### 2.1. General Results Using Wave Packets

**Figure 1:**
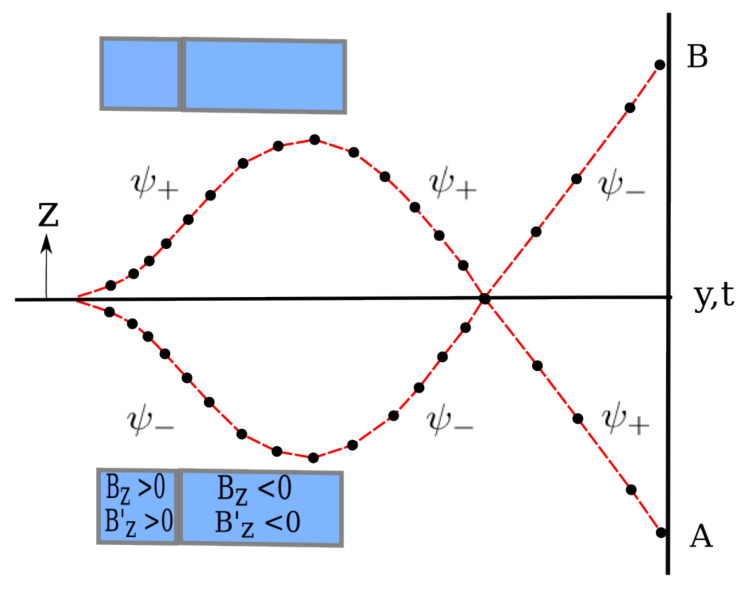
Sketch of Particle Tracks Presented in ESSW.

The ESSW paper [19] contains an error in their analysis of the Stern-Gerlach experiment as shown in Figure 1 which is similar to the set-up shown in Figure 4 appearing in ESSW [19]. It depicts the tracks of spin one-half particles entering two Stern-Gerlach (SG) magnets. The particles enter along the *y*-axis with their spins initially pointing along this axis. The orientation of the magnetic field in each SG magnet is as shown in the figure, the second SG magnet being twice the length of the first.

On entering the first magnet, the wave packet begins to split into two wave packets which move apart in the magnetic field. The packet, $\psi_{+}$, moves in the +z direction while the other, $\psi_{-}$, moves in the −z direction. Thus the $\psi_{+}$ packet follows the upper track, while the $\psi_{-}$ packet follows the lower track. Note here it is the *wave packet* we are discussing, *not* the particle.

To account for the *z*-motion of the packets, we use standard quantum mechanics as in ESSW [19], where the spin-dependent Hamiltonian is

$$\begin{array}{c} H=\frac{1}{2m}P^{2}+\varepsilon \left(t\right)\sigma_{z}-F\left(t\right)z\sigma_{z}, \end{array}$$

where $\varepsilon \left(t\right)\sigma_{z}$ is the magnetic energy at z=0 and $F\left(t\right)z\sigma_{z}$ is the energy due to the inhomogeneous field. The two components of the wave function are initially chosen to be

$$\begin{array}{c} \psi_{+}\left(z,0\right)=\psi_{-}\left(z,0\right)={\left(2\pi \right)}^{-1/4}{\left(2\delta z_{0}\right)}^{-1/2}\exp\left[-{\left(\frac{z}{2\delta z_{0}}\right)}^{2}\right], \end{array}$$

where $\delta z_{0}$ is the initial spread in *z* which is assumed small compared with the eventual maximum separation of the two beams.

At a later time, the equations of motion of the two wave packets are

$$\begin{array}{ccc} \psi_{\pm }\left(z,t\right) & = & A\left(t\right)\exp\left[-B\left(t\right){\left[z\mp \Delta z\right]}^{2}\pm \frac{i}{\hbar }\left[z\Delta p+\frac{\hbar }{2}\Phi \left(t\right)\right]\right], \end{array}$$

where $A\left(t\right)={\left(2\pi \right)}^{-1/4}{\left[2\left(\delta z_{0}+i\frac{\hbar t}{2m\delta z_{0}}\right)\right]}^{-1/2}$ and $B\left(t\right)=\frac{1}{4\delta z_{0}\left(\delta z_{0}+\frac{i\hbar t}{2m\delta z_{0}}\right)}$. In arriving at this expression we have used the impulse approximation as presented in Bohm [24]. Here $\Delta p\left(t\right)=\int_{0}{}^{t}dt^{\prime }F\left(t^{\prime }\right)$ is the momentum transferred to the “up” wave packet. The actual magnitude is not relevant to our discussion; the interested reader is referred to the original ESSW paper for these details. The magnitude of $\Phi \left(t\right)=2/\hbar \int_{0}{}^{t}dt^{\prime }\varepsilon \left(t^{\prime }\right)$ is again not relevant to our argument.

Since no measurement has been made and the two beams are still coherent, the wave function after it has traversed the magnet is written in the form

(1) $$\begin{array}{c} |\Psi \rangle =|\psi_{+}\rangle |+z\rangle +|\psi_{-}\rangle |-z\rangle . \end{array}$$

This gives the final probability density as

$$\begin{array}{c} \rho \left(z,t\right)=|\psi_{+}{\left(z,t\right)|}^{2}+{\left|\psi_{-}\left(z,t\right)\right|}^{2}, \end{array}$$

showing that there is no interference as the wave packets no longer overlap.

The *z*-component of the current is given by

(2) $$\begin{array}{cc} j(z,t) & =\frac{\hbar }{2im}\left[\Psi^{*}\frac{\partial }{\partial z}\Psi -\Psi \frac{\partial }{\partial z}\Psi^{*}\right]\\ & =\frac{\hbar }{m}\left[\left(\psi_{+}^{*}\psi_{+}+\psi_{-}^{*}\psi_{-}\right)C\left(t\right)z+\left(\psi_{+}^{*}\psi_{+}-\psi_{-}^{*}\psi_{-}\right)\left(C\left(t\right)\Delta z+\frac{\Delta p}{\hbar }\right)\right] \end{array}$$

where $C\left(t\right)=-\hbar t/[2m\left({\left(\delta z_{0}\right)}^{4}+{\left(\hbar t/2m\right)}^{2}\right)]$. Note that the probability density is symmetric about the z=0 plane, while $\left(\psi_{+}^{*}\psi_{+}-\psi_{-}^{*}\psi_{-}\right)$ is anti-symmetric, showing that the probability current is therefore antisymmetric, therefore,

(3) $$\begin{array}{c} \rho (z,t)=\rho (-z,t)\;\mathrm{with}\;j(z,t)=-j(-z,t). \end{array}$$

Also, as $\left(\psi_{+}^{*}\psi_{+}-\psi_{-}^{*}\psi_{-}\right)=0$ on the z=0 plane, j(z,t)=0 at z=0. Until this stage we agree totally with the calculations of ESSW using standard quantum mechanics based on conventional wave packet calculations, but it should be noted that this argument only holds when the incident spin is in the *y*-direction as in the ESSW thought experiment. Particle trajectories have not been discussed so far.

### 2.2. What Can Be Said about the Behaviour of Individual Particles?

Now we turn to consider what can be inferred about the behaviour of the individual particles, if anything. To answer this question let us return to Landau and Lifshitz [25] who argue that although we cannot talk about a precise particle trajectory, we can talk about the probability of finding a particle in a volume ΔV, provided the volume is large enough so that we avoid any problems associated with the uncertainty principle. Particles will flow into and out of the volume by crossing the boundary of the small volume. In this process we must ensure that probability is conserved.

To see how this works in detail, let us write the well-known conservation of probability equation in integral form. Thus

(4) $$\begin{array}{c} \frac{d}{dt}\int {\left|\Psi \right|}^{2}dV=-\int \nabla .jdV=-\oint jd\Sigma \end{array}$$

where at the last stage we have used Stokes’ theorem. Here j is the probability current density used to ensure probability conservation. The integral of this current over the surface Σ is the probability that a particle will cross the surface in unit time. By considering a series of connected volumes we can construct what can be regarded as a “macroscopic particle track”. Mott [26] has given a deeper analysis of this process.

Let us now apply this analysis to the situation shown in Figure 1. Construct a surface Σ comprising the z=0 plane and a surface enclosing the upper half of the figure so as to include the upper parts of the magnet. Since the current density is zero everywhere on the z=0 plane, no particles can cross this plane. Thus the particles that arrive in the upper-half of the experimental setup must remain in the upper-half and can never cross the z=0 plane as long as the wave packets remain coherent. This clearly shows that the continuation of the trajectories sketched in Figure 4 of the ESSW paper (as in Figure 1 here) is *not* correct.

In Figure 5 of their paper, ESSW show more explicitly the spin directions together with a sketch of two Bohm trajectories. This shows that their spin wave packets cross the z=0 axis whereas the Bohm trajectories do not. ESSW take this to mean that at first, part of the Bohm trajectories follow one of the wave packets and then, after their spin wave packets cross this axis, the trajectories follow the other wave packet. We will show in Section 4.3 the behaviour of their wave packets is not correct because they have not included spin correctly into the Bohm model.

## 3. The Bohm Approach When Spin Is Included

To give an account of the behaviour of a particle with spin in the non-relativistic limit, we must widen the scope of the Bohm approach. An extended model for a spin-half particle based on the Pauli equation has already been presented in Bohm, Schiller and Tiomno (BST) [27]. Full details of this model have also been discussed in a series of papers by Dewdney et al. [28,29,30,31] and by Holland [32]. This simple model has been applied to neutron diffraction and a single Stern-Gerlach magnet, the results being reported in [29,30]. It should be noted that none of this work is referred to in the ESSW paper and yet this is clearly significant as the Stern-Gerlach magnets operate on the magnetic moments of the particles.

If they had been aware of this work they would not have made the statement that in the Bohm theory a particle has a position and *nothing else*. In the BST extension, not only do we have position, but also the orientation of the spin vector. Here the Euler angles (θ,ϕ,ψ) are used to specify the spin direction. This is essentially the precursor of the flag picture of the spinor presented in Penrose and Rindler [33]. Bell [34] has a simpler model which was also based on the three components of the spin vector. A more general approach using Clifford algebras in which the Pauli spin matrices play a fundamental role has been presented in Hiley and Callaghan [35]. This approach shows how the BST model emerges as a particular representation using Euler angles.

### 3.1. Spin and the Use of the Pauli Equation

We start with the Pauli equation

(5) $$\begin{array}{c} i\hbar \frac{\partial \xi }{\partial t}=H\xi , \end{array}$$

where ξ is the two-component spinor which we write in the form

(6) $$\begin{array}{c} \xi =Re^{i(\psi /2)}\left(\begin{array}{c} \cos\left(\theta /2\right)e^{i(\phi /2)}\\ i\sin\left(\theta /2\right)e^{-i(\phi /2)} \end{array}\right). \end{array}$$

Here (θ,ϕ,ψ) are the three Euler angles.

The Hamiltonian *H* is then written in the form

(7) $$\begin{array}{c} H=-\frac{\hbar^{2}}{2m}{\left(\nabla -\frac{ie}{2m}A\right)}^{2}+\mu \sigma .B+V, \end{array}$$

where μ is the magnetic moment of the particle.

The original physical idea here was to assume the particle is a spinning object whose orientation is specified by the three Euler angles (θ,ϕ,ψ). The probability of the particle being at a given position, (r,t), is $\rho \left(r,t\right)=R^{2}\left(r,t\right)={\left|\xi \left(r,t\right)\right|}^{2}$. This means the properties of the Pauli particle are specified by four real numbers (ρ,θ,ϕ,ψ) given at the point (r,t). The time evolution of these parameters is determined by the Pauli Equation (5) as we will now show.

It is more convenient to rewrite the wave function in the form

$$\begin{array}{c} \xi \left(r,t\right)=\left(\begin{array}{c} R_{+}e^{i\frac{S_{+}}{\hbar }}\\ R_{-}e^{i\frac{S_{-}}{\hbar }} \end{array}\right), \end{array}$$

where

(8) $$\begin{array}{c} \theta =2\tan^{-1}\frac{R_{-}}{R_{+}};\;\psi =\frac{S_{+}+S_{-}}{\hbar }-\pi /2;\;\phi =\frac{S_{+}-S_{-}}{\hbar }+\pi /2. \end{array}$$

To find the velocity of the particle, let us first write the quantity $\xi^{†}\nabla \xi$ in terms of the Euler angles,

$$\begin{array}{c} \xi^{†}\nabla \xi =R\nabla R+\frac{i}{2}R^{2}\nabla \psi +\frac{i}{2}\cos\theta R^{2}\nabla \phi . \end{array}$$

Then following Hiley [36] we can define a complex local velocity

$$\begin{array}{c} v=v_{Re}+iv_{Im}=\frac{-i\hbar }{m}\frac{\xi^{†}\nabla \xi }{\xi^{†}\xi } \end{array}$$

where the probability density is given by $R^{2}=\xi^{†}\xi$.

The real part of the local velocity is

(9) $$\begin{array}{c} v_{Re}=\frac{\hbar }{2m}\hat{z}\left(\nabla \psi +\cos\theta \nabla \phi \right) \end{array}$$

which replaces v(r,t)=∇S(r,t)/m defined for the spin-less particle. The imaginary part, which was not discussed by Bohm in his original paper (but see Bohm and Hiley [37]) is called the “osmotic velocity” and has the form

(10) $$\begin{array}{c} v_{Im}=-\frac{i\hbar }{m}\hat{z}\left[\frac{\nabla R}{R}\right]. \end{array}$$

We will now use Equations (9) and (10) to simulate the detailed behaviour of the particles and their spin orientations as they traverse the set-up illustrated in Figure 1.

## 4. Detailed Calculation of the Trajectories

### 4.1. One Stern-Gerlach Magnet

We begin by simulating the behaviour of the particles having passed through a single Stern-Gerlach magnet. For simplicity we use the impulse approximation given in Bohm [24] to analyse the evolution of a wave packet as it leaves the magnet (A full treatment using Feynman propagators is being prepared by Hiley and Callaghan. This allows us to calculate trajectories inside the SG. Preliminary results confirm the results presented here.).

In the Hamiltonian given in Equation (7), we replace *B* by the field in the SG magnet, which we write as $B\approx \mu (B_{0}+zB_{0}^{\prime })$, where $B_{0}^{\prime }$ is the field gradient inside the magnet and set A and *V* to zero.

Following Dewdney et al. [30] and Holland [32], we choose the initial wave function to be

$$\begin{array}{c} \xi_{0}=\xi_{+}+\xi_{-}=f\left(z\right)\left(c_{+}u_{+}+c_{-}u_{-}\right)={\left(2\pi \right)}^{-1/2}\int g\left(k\right)\left(c_{+}u_{+}+c_{-}u_{-}\right)e^{ikz}dk, \end{array}$$

where $g\left(k\right)={\left(2\sigma^{2}/\pi \right)}^{1/4}e^{-k^{2}\sigma^{2}}$ is a normalised Gaussian packet centred at k=0 in momentum space. Here $u_{+}$ and $u_{-}$ are the eigenstates of the spin operator $\sigma_{z}$. The solution of the Pauli equation at time *t* after the particle has left the SG magnetic field is

$$\begin{array}{c} \xi ={\left(2\pi \right)}^{-1/2}\int dkg\left(k\right)\{c_{+}u_{+}\exp\left[i\left(-\Delta +\left(k-\Delta^{\prime }\right)z-\frac{\hbar t}{2m}{\left(k-\Delta^{\prime }\right)}^{2}\right)\right]\\ +c_{-}u_{-}\exp\left[i\left(\Delta +\left(k+\Delta^{\prime }\right)z-\frac{\hbar t}{2m}{\left(k+\Delta^{\prime }\right)}^{2}\right)\right]\} \end{array}$$

where $\Delta =\mu B_{0}\Delta t/\hbar ,\Delta^{\prime }=\mu_{0}B_{0}^{\prime }\Delta t/\hbar$ and Δt is the time spent in the field. Carrying out the integral we find

(11) $$\begin{array}{c} \xi \left(z,t\right)={\left(2\pi s_{t}^{2}\right)}^{-1/4}\{c_{+}u_{+}\exp\left[-{\left(z+ut\right)}^{2}/4\sigma s_{t}\right]\exp\left[-i(\Delta +\left(z+\frac{1}{2}ut\right)\Delta^{\prime })\right]\\ +c_{-}u_{-}\exp\left[-{\left(z-ut\right)}^{2}/4\sigma s_{t}\right]\exp\left[i(\Delta +\left(z-\frac{1}{2}ut\right)\Delta^{\prime })\right]\}. \end{array}$$

Here $s_{t}=\sigma \left(1+i\hbar t/2m\sigma^{2}\right)$, and $u=\hbar \Delta^{\prime }/m.$ We now write ξ(t) in the form

(12) $$\begin{array}{c} \xi \left(z,t\right)=c_{+}R_{+}e^{iS_{+}/\hbar }u_{+}+c_{-}R_{-}e^{iS_{-}/\hbar }u_{-} \end{array}$$

where

(13) $$\begin{array}{c} R_{\pm }={\left(2\pi \sigma^{2}\right)}^{-1/4}{\left(1+\hbar^{2}t^{2}/4m^{2}\sigma^{4}\right)}^{-1/4}\exp\left(\frac{-{\left(z\pm ut\right)}^{2}}{4\sigma^{2}\left(1+\hbar^{2}t^{2}/4m^{2}\sigma^{4}\right)}\right) \end{array}$$

and

(14) $$\begin{array}{c} S_{\pm }/\hbar =\mp \Delta \mp \left(z\pm \frac{1}{2}ut\right)\Delta^{\prime }-\frac{1}{2}\tan^{-1}\left(\hbar t/2m\sigma^{2}\right)+\frac{\hbar t{\left(z\pm ut\right)}^{2}}{8m\sigma^{4}\left(1+\hbar^{2}t^{2}/4m^{2}\sigma^{4}\right)}. \end{array}$$

We are now in a position to calculate the local velocities from the specific solution given by Equation (12). Since the real part of the local velocity is given by Equation (9), namely, ℏ(∇ψ+cosθ∇ϕ)/2m, we need only evaluate ∂ψ/∂z and ∂ϕ/∂z since we are only considering the motion along the *z*-direction. In order to find these derivatives, and those required for the osmotic velocity and the quantum potential, we express the parameters (ρ,θ,ϕ,ψ) in terms of $\left(R_{+},R_{-},S_{+},S_{-}\right)$ using Equations (8), (13) and (14), and obtain,

$$\begin{array}{c} \frac{\partial \psi }{\partial z}=\frac{4\hbar tz}{8m\sigma^{4}\left(\hbar^{2}t^{2}/4m^{2}\sigma^{4}+1\right)}, \end{array}$$

$$\begin{array}{c} \frac{\partial \phi }{\partial z}=-2\Delta^{\prime }+\frac{4\hbar ut^{2}}{8m\sigma^{4}\left(\hbar^{2}t^{2}/4m^{2}\sigma^{4}+1\right)}, \end{array}$$

$$\begin{array}{c} \frac{\partial \theta }{\partial z}=\sin\theta \frac{ut}{\sigma^{2}\left(\hbar^{2}t^{2}/4m^{2}\sigma^{4}+1\right)}, \end{array}$$

and

$$\begin{array}{c} \frac{1}{R}\frac{\partial R}{\partial z}=-\frac{z+ut\cos\theta }{2\sigma^{2}\left(\hbar^{2}t^{2}/4m^{2}\sigma^{4}+1\right)}. \end{array}$$

The Bohm velocity given by Equation (9) then becomes

(15) $$\begin{array}{c} v_{Re}=\frac{\hbar \hat{z}}{2m}\left(-2\Delta^{\prime }\cos\theta +\frac{\hbar t[z+ut\cos\theta ]}{2m\sigma^{4}\left(\hbar^{2}t^{2}/4m^{2}\sigma^{4}+1\right)}\right). \end{array}$$

Note here that the second term in the above expression corresponds to the spreading of the wave packet and contributes little to the overall behaviour. The main effect of the field comes from the first term $\Delta^{\prime }\cos\theta$, which reveals clearly how the velocities and therefore the trajectories are strongly affected by the behaviour of the spin vector. This term depends implicitly on (z,t,u) and is responsible for the splitting of the beam.

The imaginary part or osmotic local velocity given in Equation (10), namely, $v_{Im}=-i\hbar \left[\nabla R/R\right]/m$, now becomes

(16) $$\begin{array}{c} v_{Im}=\frac{2\hbar }{m}\frac{\hat{z}\left[z+ut\cos\theta \right]}{\sigma^{2}\left(\hbar^{2}t^{2}/4m^{2}\sigma^{4}+1\right)}. \end{array}$$

Note there is no explicit dependence on the magnetic field gradient but there is an implicit dependence through *u* and cosθ.

These results enable us to calculate specific trajectories and spin vectors for various particle initial positions and for various values of $\left(c_{+},c_{-}\right)$ should that become necessary. The choice of the latter determine the initial value of the spin vector direction θ which, in our case was chosen to be along the *y*-direction, hence $\left(c_{+},c_{-}\right)=1/\sqrt{2}$. The results shown in figures below are calculated for parameters listed in Table 1.

### 4.2. Numerical Values for Single Stern-Gerlach Magnet

**Figure 2:**
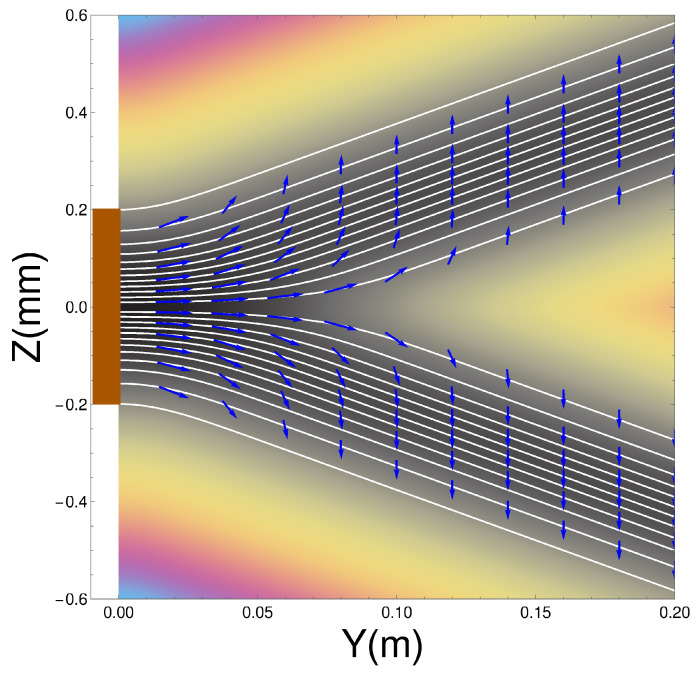
Trajectories with spin vectors immediately on exiting the Stern-Gerlach (SG) magnet.

Integrating Equation (9) will give us the Bohm trajectories. In Figure 2 we show the ensemble of Bohm trajectories and the spin orientations as they leave the Stern-Gerlach magnet, shown in brown at the LHS of the figure. The background colours show the probability density, black being the greatest, while blue is zero.

The dark background shows how the wave packets diverge along straight lines, as do the trajectories. Superimposed on the trajectories are the spin orientations.

Notice that, contrary to the conventional view, the atoms do not immediately “jump” into one or other *z*-spin eigenstates, rather the spin vectors undergo continuous evolution until they reach their final *z*-spin eigenstate. This occurs once the two wave packets $\psi_{+}\left(z,t\right)$ and $\psi_{-}\left(z,t\right)$ have separated and have no significant overlap. The upper beam will contain only atoms with spin “up" in the *z*-direction while those in the lower beam will all be “down” in the *z*-direction. Notice also that the rotational changes occur in a *magnetic field-free region*. We can also see that the alignment of the spin vector at a given *y* value close to the magnet depends on *z*, with the spin associated to trajectories closer to the z=0 axis rotated least. In Section 4.7 we will see that the cause of these behaviours is a torque produced by the quantum potential. These results for a single magnet confirm what was already found in Dewdney et al. [29,30,31].

**Figure 3:**
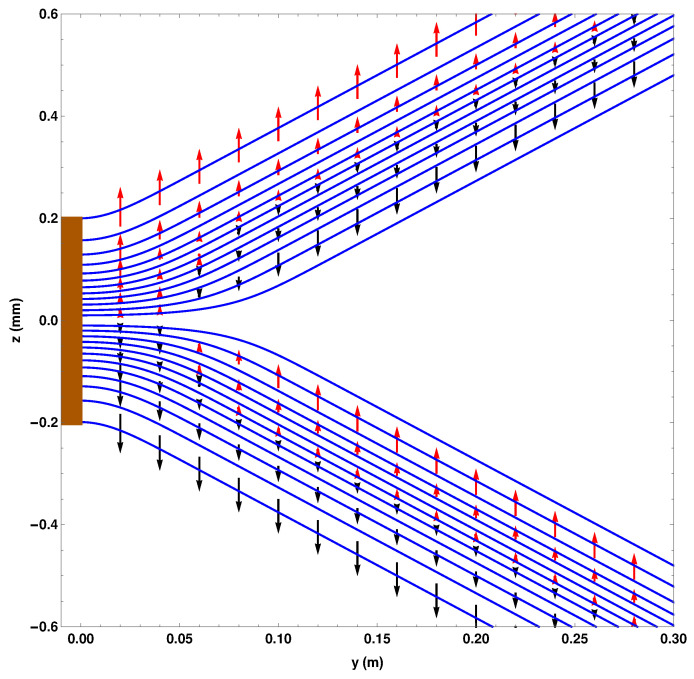
The osmotic flow vectors immediately on exiting the SG magnet.

Figure 3 shows the effect of the osmotic velocity, which we have represented by arrows. They are responsible for maintaining the wave packet profile and will be discussed further in Section 4.6.

### 4.3. Two Stern-Gerlach Magnets

Having seen how the atoms behave in a single SG magnet, let us now move on to consider two SG magnets with opposite field directions as shown in Figure 1. Note here the second SG magnet is double the length of the first.

The method is similar to the case of the single magnet, except now we use, as initial wave packet, the inverse Fourier transform of the wavefunction at the second magnet at time $t=t_{1}$. We obtain the real part of the local velocity as

(17) v2Re=ℏz^2m−2Δ2′−2Δ1′ℏ2(t1+t)24m2σ4+1cosθ+ℏ(t1+t)2mσ4ℏ2(t1+t)24m2σ4+1z+u2tcosθ

and the osmotic velocity as

**Figure 4:**
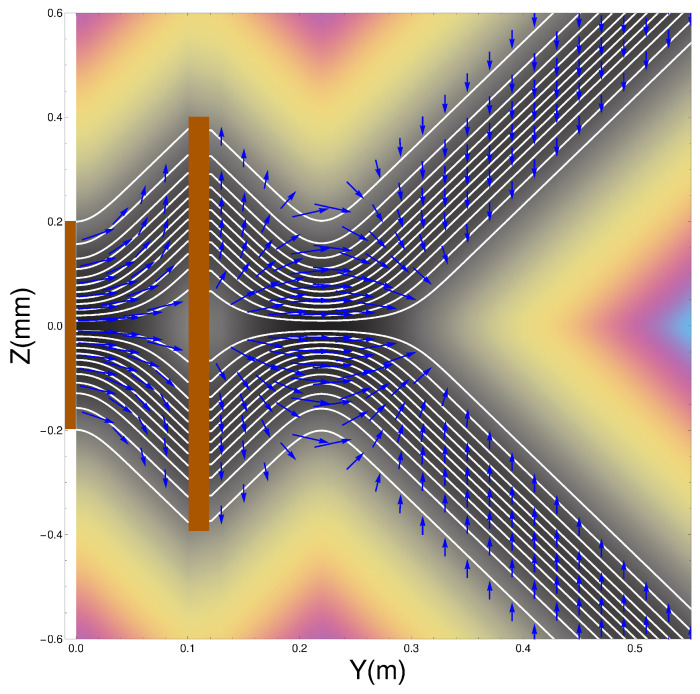
Spins emerging from two Stern-Gerlach magnets.

(18) v2Im=ℏz^m12σ2ℏ2(t1+t)24m2σ4+1z+u1(t1+t)+u2tcosθ

where t=0 at the exit of the second magnet. In Figure 4 we have plotted the trajectories together with the spin orientations as the atoms pass through two SG magnets. The details of the parameters used in the calculations are again as listed in Table 1. The position of the second magnet is as indicated by the brown bar between y=0.1 m and y=0.12 m.

There are several features of the ensemble of trajectories that are noteworthy. Firstly, at the exit of the second magnet, the wave-packets are refocused toward the *y*-axis until the inner edge of the packets reaches the axis at y≈0.22 m at which point they diverge again.

Secondly, no trajectories are found to cross the *z* = 0 plane. This should, in fact, not be surprising since $v_{Re}$ can also be obtained from j(z,t)/ρ(z,t). This means that the “Bohm trajectories” are identical to the probability flow lines and, as we have seen, the probability flow lines do not cross the z=0 plane. Thus there is no experimental difference between the Bohm approach and standard quantum mechanics at this stage. It could be argued that it is quantum mechanics that is “at variance with common sense”!

Thirdly, notice once again that the spins do not immediately “jump” into the eigenstates as assumed by the standard theory. Rather they take a small but finite time to reach the final eigenstate as discussed above in Section 3.1. Furthermore note that when the beams are refocused close to the *z* = 0 plane, at about y=0.22, the spin vectors are rotated so that they all become aligned with the *y*-axis before being rotated again until they end up anti-parallel to the direction with which they entered the second magnet. This rotation is very surprising but is generated by the quantum torque that arises from the quantum potential as we show in the next section in Equation (21).

**Figure 5:**
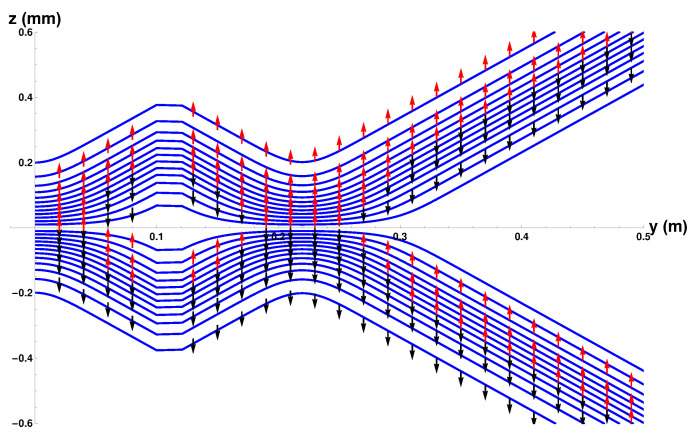
The osmotic velocity superimposed on the trajectories for two Stern-Gerlach magnets.

Furthermore this is in contradiction with Figure 5 of ESSW where they argue that the Bohm trajectories are not realistic because in order to get the observed final spin state, their particles must cross the z=0 axis. Therefore the present work shows clearly the importance of coupling the spin and the centre of mass motion in order to obtain a correct and consistent analysis of the problem.

Figure 5 shows the direction of the osmotic velocity in the two SG magnets case. Its behaviour is again exactly the same as in the one SG magnet case.

To return the packet to its original state with all the spins pointing in the *y*-direction, we have to add a third magnet as indicated in the original diagram in Feynman et al. [38]. Thus the Bohm approach gives a complete account of the average behaviour of the individual quantum processes.

### 4.4. The Appearance of the Quantum Torque

Now let us show the source of the quantum torque. We start by examining the real part of the Pauli Equation (5) under polar decomposition of the wave function, which can be written in the form

(19) $$\begin{array}{c} \frac{1}{2}\hbar \left(\frac{\partial \psi }{\partial t}+\cos\theta \frac{\partial \phi }{\partial t}\right)+\frac{1}{2}mv^{2}+Q_{P}+\frac{2\mu }{\hbar }\sigma .s+V=0. \end{array}$$

Here once again we see, as in the case of the Schrödinger equation, an extra energy term, $Q_{P}$, the quantum potential energy, appears. In the present case $Q_{P}$ takes the form

(20) $$\begin{array}{c} Q_{P}=-\left(\hbar^{2}\nabla^{2}R\right)/2mR-\frac{\hbar^{2}}{8m}\left[{\left(\nabla \theta \right)}^{2}+\sin^{2}\theta {\left(\nabla \phi \right)}^{2}\right]. \end{array}$$

The first term will be recognised as the quantum potential found in the Schrödinger equation. The second term determines the evolution of the spin vector which is given by

$$\begin{array}{c} s=\frac{1}{2}\hbar \xi^{†}\sigma \xi =\frac{1}{2}\left(\sin\theta \sin\phi ,\sin\theta \cos\phi ,\cos\theta \right). \end{array}$$

The equation of motion for the spin vector s, is then found to be

(21) $$\begin{array}{c} \frac{ds}{dt}=T-\frac{2\mu }{\hbar }\left(s\times B\right). \end{array}$$

Here B is an external magnetic field and

(22) $$\begin{array}{c} T={\left(m\rho \right)}^{-1}s\times \sum_{i}\frac{\partial }{\partial x_{i}}\left(\rho \frac{\partial s}{\partial x_{i}}\right). \end{array}$$

It is the quantum torque, T, that acts on the individual atoms, rotating their spin vectors and the flag plane.

### 4.5. Detailed Calculation of the Quantum Potential

To understand better the role played by the quantum potential, let us examine in more detail its mathematical structure as shown in Equation (20). We restrict our analysis to the case of a single magnet. As the quantum Hamilton-Jacobi Equation (19) is an equation that conserves energy, the appearance of *Q* implies that some of the kinetic energy of the particle is transferred to the quantum potential energy *Q*. As we see from Equation (20), the quantum potential energy has two components

$$\begin{array}{c} Q_{trans}=-\frac{\hbar^{2}\nabla^{2}R}{2mR}\;\mathrm{and}\;Q_{spin}=\frac{\hbar^{2}}{8m}\left[{\left(\nabla \theta \right)}^{2}+\sin^{2}\theta {\left(\nabla \phi \right)}^{2}\right]. \end{array}$$

We will examine the two terms independently. First consider $Q_{trans}$. Since the particle is moving in one-dimension

$$\begin{array}{c} \nabla^{2}R\to \frac{\partial^{2}R}{\partial z^{2}}=-\frac{2}{bd}\left(-\frac{2R{\left(z+ut\cos\theta \right)}^{2}}{bd}+R\left(1-\frac{4u^{2}t^{2}}{bd}\right)\sin^{2}\theta \right), \end{array}$$

where we have written

$$\begin{array}{c} b=\left(\frac{\hbar^{2}t^{2}}{4m^{2}\sigma^{4}}+1\right)\;\mathrm{and}\;d=4\sigma^{2}. \end{array}$$

Then

$$\begin{array}{c} Q_{trans}=-\frac{\hbar^{2}\nabla^{2}R}{2mR}=\frac{\hbar^{2}}{bdm}\left(-\frac{2}{bd}\left[{\left(z+ut\cos\theta \right)}^{2}+2u^{2}t^{2}\sin^{2}\theta \right]+1\right). \end{array}$$

Now we turn to evaluate the spin part of the quantum potential, $Q_{spin},$ where we need to evaluate

$$\begin{array}{c} \nabla \phi =-2\Delta^{\prime }+\frac{4\hbar ut^{2}}{8mb\sigma^{4}}\;\mathrm{and}\;\nabla \theta =\sin\theta \frac{ut}{b\sigma^{2}}. \end{array}$$

This gives

$$\begin{array}{c} Q_{spin}=\frac{\hbar^{2}\sin^{2}\theta }{8bm}\left(\frac{u^{2}t^{2}}{\sigma^{4}}-2\Delta^{\prime }\frac{\hbar ut^{2}}{2m\sigma }+4\Delta^{\prime 2}\left(\frac{\hbar^{2}t^{2}}{4m^{2}\sigma^{4}}+1\right)\right). \end{array}$$

The expression for the total quantum potential, $Q=Q_{trans}+Q_{spin}$ is rather complex so it will be helpful if we can make an approximation without significantly altering the final result. This can be done by noticing the magnitude of $b=\left(\frac{\hbar^{2}t^{2}}{4m^{2}\sigma^{4}}+1\right)\approx 1$. This means that we are assuming the wave packet does not spread significantly during the flight times considered. We arrive at the final expression for the total quantum potential:

$$\begin{array}{c} Q\approx \frac{\hbar^{2}}{m\sigma }\left(-\frac{2}{m\sigma^{4}}\left[{\left(z+ut\cos\theta \right)}^{2}+2u^{2}t^{2}\sin^{2}\theta \right]+1\right)\\ +\frac{\hbar^{2}\sin^{2}\theta }{8m}\left(\frac{u^{2}t^{2}}{\sigma^{4}}-2\Delta^{\prime }\frac{\hbar ut^{2}}{m\sigma^{4}}+4\Delta^{\prime 2}\right). \end{array}$$

### 4.6. Numerical Details: Quantum Potential Single Stern-Gerlach Magnet

**Figure 6:**
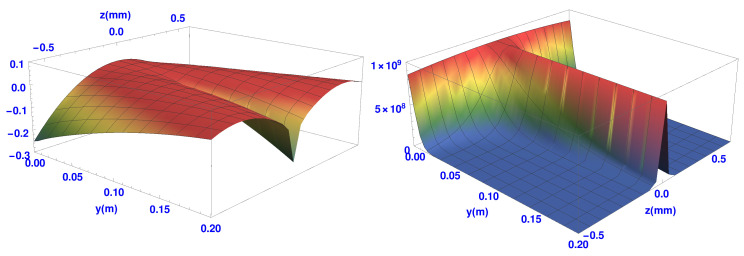
Transverse (**left**) and spin (**right**) quantum potential at exit of a single SG magnet.

In Figure 6 below we plot the transverse quantum potential $Q_{trans}$ and the spin quantum potential, $Q_{spin}$ for the single SG magnet. The end of the SG magnet is again along the *z*-axis at *y* = 0, with the atoms flowing along the *y*-axis out of the page.

The atoms initially experience the first part of the quantum potential where the beam begins to split into two as shown in Figure 2. Both quantum potentials split symmetrically into two parts about the *y*-axis. The two “domes” of $Q_{trans}$, shown in the left hand of the figure, cover each beam as they separate. The width of each dome characterises the spreading wave packet as it evolves in time. Also, when compared to the osmotic velocities shown in Figure 3, we can see how these velocities are related to the gradient of $Q_{trans}$. The trajectories are seen to follow paths of constant gradient and the osmotic velocities are constant along the trajectories in Figure 3. Furthermore, those trajectories in the wings of the wave packets experience a more steep gradient and the osmotic velocities are indeed found to be larger there. At the maximum of the packet, the osmotic velocity is zero. An interpretation of the $Q_{trans}$ would therefore be that it gives rise to a force, which is anti-parallel to the osmotic velocity and restricts the spreading of the wave packet.

**Figure 7:**
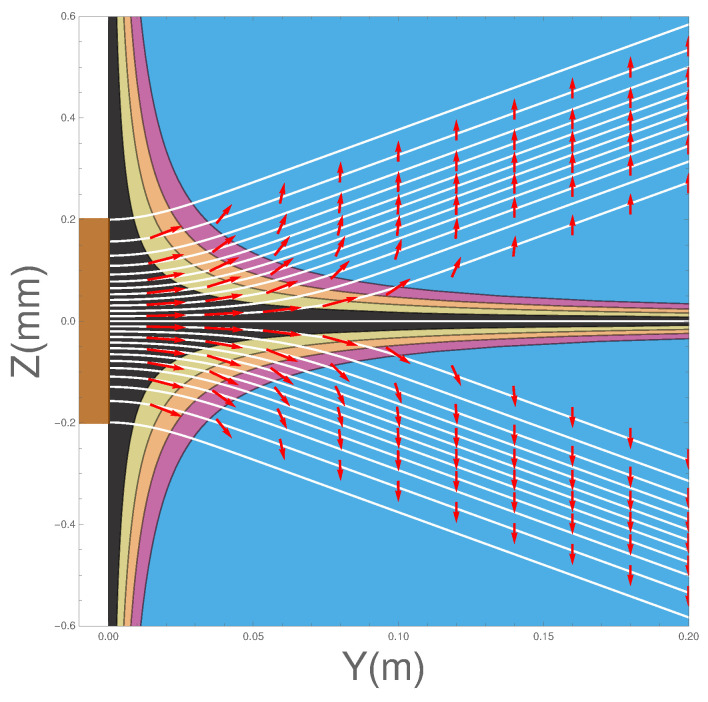
Trajectories with spin vectors overlaid on the spin quantum potential immediately on exiting a single SG magnet.

The spin part of the quantum potential $Q_{spin}$ is shown in the right hand of Figure 6. The upward slope produces the quantum torque that rotates the spin vectors of the atoms as the two beams separate. This rotation continues until the two packets are completely separate. When this happens all the spins point “up” in the upper beam, while they all point “down” in the lower beam. At this stage the $Q_{spin}\to 0$ ensuring the atoms remain in their final spin eigenstates. Figure 7 shows the projection of the $Q_{spin}$ of Figure 6 on the trajectories and spin orientation. Note also that the trajectories close to the *y*-axis do not experience the same steepness of $Q_{spin}$ as do those which are off-axis. This explains why, as remarked earlier, the spin vectors closer to the *y*-axis take longer to align themselves either up or down.

### 4.7. Numerical Details: Quantum Potential in Two SG Magnet Case

Now let us consider the case when the two Stern-Gerlach magnets are in place. The positions of the magnets are shown in brown. Recall here that the inhomogeneities in the magnetic fields oppose each other.

**Figure 8:**
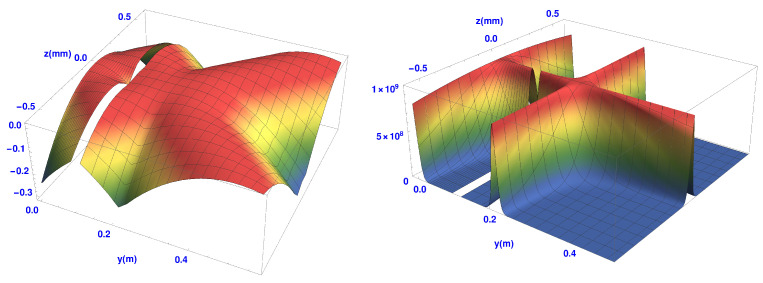
$Q_{trans}$ (**left**) and $Q_{spin}$ (**right**) quantum potential for a two SG magnets system.

In Figure 8 we show both $Q_{trans}$ and $Q_{spin}$ for the case of two magnets. The gap in each figure corresponds to the position of the second magnet. The quantum potential after the second magnet is similar to that of the single SG magnet as shown in Figure 6. These results give a detailed picture of the expected evolution of a non-relativistic atom with spin one-half as it goes through both SG magnets.

**Figure 9:**
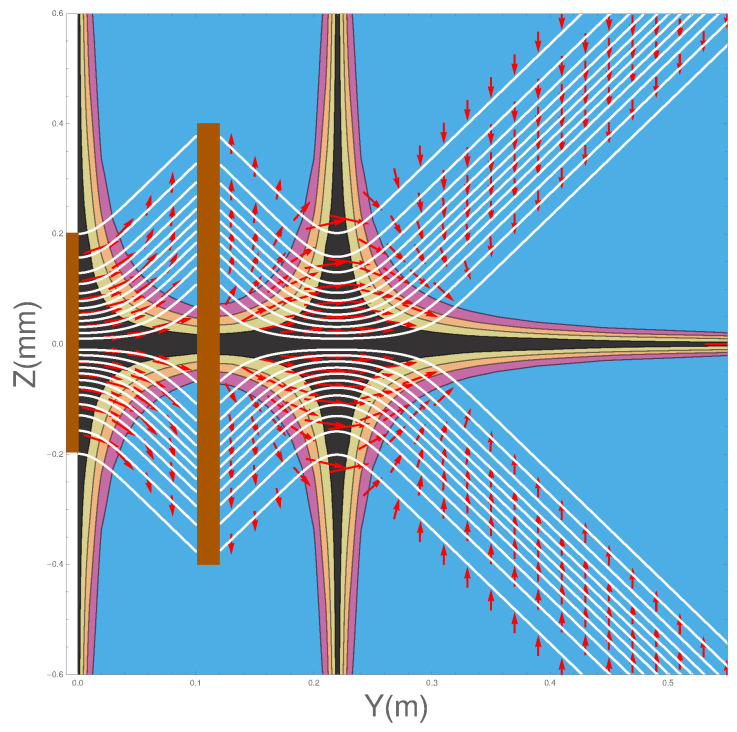
Trajectories with spin vectors overlaid on the spin quantum potential for a two SG magnet system.

Figure 9 shows the projection of the spin quantum potential superimposed on the trajectories. Notice that the quantum torque is strongest well outside the second SG magnet in the magnetic field-free region, producing a 180 degree rotation of the spin vector. It is at this point that the wave packets begin to interfere strongly. In fact the quantum torque continues to act outside the magnet until the two wave packets $\psi_{+}\left(z,t\right)$ and $\psi_{-}\left(z,t\right)$ cease to overlap. Notice once again how the spin does not immediately ‘jump’ into one of the two spin *z*-eigenstates, but undergoes a well-defined time evolution. Such a behaviour would have, perhaps, been welcomed by Schrödinger himself [39].

Once they no longer overlap, each atom remains in one or the other spin eigenstates. Again, as was the case with the single SG magnet, the spin vectors along the trajectories close to the *y*-axis, especially at the point where the two beams are refocused, experience less of the gradient of $Q_{spin}$. Thus it is clear that the quantum torque arises from the interference region, implying it is an internal feature of the overall behaviour, suggesting a kind of dramatic re-structuring of the underlying process.

Bohm was intuitively well aware of this possibility and it was one of the reasons why he abandoned the view that the atom only had a local, “rock-like” property. He preferred to regard the atom as a quasi-local region of energy undergoing a new type of process that he described in more general terms as an “unfolding-enfolding” process, comparing it to a gas near its critical point, the particle itself constantly forming and dissolving, as in critical opalescence [40,41]. In other words, the quantum evolution involves an entirely new re-ordering process which should not be regarded as a particle following a well defined trajectory.

This view of the evolving quantum process becomes even more compelling since Hiley and Callaghan [42] and Takabayasi [43] have shown that the local momentum and energy are actually related to the energy-momentum tensor, $T^{\mu \nu }$, through the relations

$$\begin{array}{c} \rho p^{j}\left(r,t\right)=T^{0j}\left(r,t\right)\;\mathrm{and}\;\rho E\left(r,t\right)=T^{00}\left(r,t\right), \end{array}$$

a feature of which Schwinger [44] was well aware. The question of which particular trajectory a specific atom actually takes cannot be answered because the experimenter has no way of choosing or controlling the initial position of the particle. The final result is also totally independent of the observer. A detailed discussion of the role of the experimenter in the Bohm approach can be found in Bohm and Hiley [45,46]. A more recent paper by Flack and Hiley [47] shows how the Bohm trajectories emerge from an averaging over this deeper process.

We can see from Figure 9, the above simulations predict some interesting structure in near field behaviour of the atoms after they leave the second SG magnet. This could be experimentally explored through weak measurements as suggested in [48]. At present, our group [49] is attempting to measure the weak values of momentum and spin which, if successful, would ultimately enable us to not only construct these flow lines, but also to measure the time evolution of the angle θ(y,t) of the spin vector.

We are also exploring the possibility of using the techniques we are developing to check the results shown in Figure 2. At present we are on the edge of what is technically possible and if we are successful, the experiments will show that the quantum potential energy appearing in Equation (19) has an observable experimental consequence and therefore cannot be ignored in analysing quantum phenomena.

## 5. Conclusions

In this paper we have shown that the differences that are claimed to exist between the standard approach to quantum mechanics and the Bohm approach do not exist when both are applied correctly. Indeed it is hard to imagine how there could be any differences in the predicted experimental results since *both* approaches use exactly the same mathematical structure. For the type of experiments considered by ESSW [19], the probability current plays a key role. In both approaches the probability current is considered as a particle flow, the conventional approach regarding it as a measure of particles flowing out of a small region, ΔV, of space, whereas the Bohm approach assumes the probability current arises from the velocities of individual particles through the relation j(r)/ρ(r)=∇S(r)/m=p(r)/m. In the Bohm model this is taken as the definition of the local momentum, p(r). Clearly the behaviour of the probability currents is identical to the local momentum. This is what ESSW failed to recognise. Notice that this disagreement arises before the addition of any device to measure which path the particle actually took.

The inclusion of a which-way detector into the discussion merely confuses the issue. Traditionally it is assumed that any measurement to determine which path a particle actually takes brings about the “collapse” of the wave function. Suppose a position measurement is made after the atom has left the second SG magnet as shown in Figure 1. The wave function (1) will not then be the pure state but instead will be a mixture which must be described by a density matrix ρ with $\rho^{2}\neq \rho$. This means there is no interference between the two wave packets $\psi_{+}$ and $\psi_{-}$ in which case the particles actually cross the z=0 plane as shown in Figure 1. Exactly the same thing happens in the Bohm model as was discussed in detail in Hiley [22] and Hiley and Callaghan [23]. We will not repeat the argument again in this paper but refer the interested reader to the original papers. Our conclusion is that the standard quantum mechanics produces exactly the same behaviour as the Bohmian approach so it cannot be used to conclude the Bohm trajectories are “surreal”.

Since these earlier objections were raised, an entirely new way of experimentally constructing the “Bohm particle trajectories” has been developed by Kocsis et al. [1] as discussed in the introduction. Furthermore in the case of atoms the claim that these are “particle trajectories” has been re-examined recently by Flack and Hiley [47] who have concluded that the flow lines, as we shall now call them, are not the trajectories of single atoms but an average momentum flow, the measurements being taken over many individual particle events. In fact they have shown that they represent an average of the ensemble of actual individual stochastic Feynman paths.

The calculations we have presented in this paper provide a detailed background to the experiments of Monachello et al. [49] and Morley et al. [50]. This means that we will not have to rely on theoretical arguments alone to reach an understanding of the behaviour reported in this paper but we hope to be able to provide experimental evidence to further clarify the situation.

## Figures and Tables

**Table 1:** Parameters used in the numerical investigation.

Atom	Ag
Mass	$1.8\times 10^{-25}$ Kg
Width of magnets	4 and $8\times 10^{-4}$ m
Length of magnets	1 and $2\times 10^{-2}$ m
Velocity of atoms	$v_{y}=y/t=500$ m/s
Time within magnets	Δt=2 and $4\times 10^{-5}$ s
Magnetic field strength at centre	$B_{0}=5$ Tesla
Magnetic field gradient	$B_{0}^{\prime }=1000$ Tesla/m
Wave packet width	$\sigma =1\times 10^{-4}$ m
Wave packet speed	$u=\mu_{B}B_{0}^{\prime }\Delta t/m=1$ m/s
$\Delta^{\prime }=\mu_{B}B_{0}^{\prime }\Delta t\hbar =mu/\hbar$	$\Delta^{\prime }=1.717\times 10^{9}$ m${}^{-1}$
